# Preclinical Assessment of a Novel Conformable Foam-Based Left Atrial Appendage Closure Device

**DOI:** 10.1155/2021/4556400

**Published:** 2021-06-10

**Authors:** Robert J. Sommer, Ronald Lamport, David Melanson, Carol Devellian, Andy Levine, Christopher M. Cain, Aaron V. Kaplan, William A. Gray

**Affiliations:** ^1^Columbia University Medical Center, NY, NY, USA; ^2^Conformal Medical, Inc., Nashua, NH, USA; ^3^Heart & Vascular Center, Dartmouth Hitchcock Medical Center, Lebanon, NH, USA; ^4^Lankenau Heart Institute, Wynnewood, PA, USA

## Abstract

**Background:**

Left atrial appendage (LAA) occlusion has been established as an alternative to systemic anticoagulation for stroke prevention in patients with atrial fibrillation; however, limitations of current devices have slowed adoption. We present preclinical evaluations of a novel device, the Conformal Left Atrial Appendage Seal (CLAAS).

**Methods:**

An in vitro assessment of conformability was conducted to evaluate the two CLAAS devices (regular 27 mm and large 35 mm) and a Watchman 2.5 (27 mm). Devices were placed within silicone tubes and compressed in a vise submerged in a water bath at 37°C. Changes in device diameter and visual seal were noted. Acute (*n* = 1) and chronic 60-day (*n* = 6) canine studies with gross and histologic assessment were performed.

**Results:**

Conformability bench tests demonstrated that the regular CLAAS implant was able to seal oval orifices from 20 × 30 mm to 15 × 33 mm and the large from 30 × 35 mm to 20 × 40 mm. As the CLAAS implant was compressed in the minor diameter, it increased in the major diameter, thereby filling the oval space, whereas the Watchman 2.5 showed gaps and maintained its round configuration when compressed in one direction. Seven devices were successfully implanted in the canine model with complete seal without thrombus. Histologic examination showed complete neointima covering with minimal inflammation at 60 days.

**Conclusions:**

Preclinical testing demonstrated the conformability of the CLAAS implant and its ability to seal the LAA. Clinical studies are ongoing to characterize the utility of the CLAAS implant in the treatment of patients with atrial fibrillation.

## 1. Introduction

Atrial fibrillation (AF) is the most common cardiac arrhythmia and affects more than 5 million patients in the United States and 33 million worldwide. AF is associated with thromboembolic stroke, accounting for approximately 20% of hospital admissions for ischemic stroke, and the lifetime stroke risk for a patient diagnosed with AF is approximately 1 in 3 [1–4]. Oral anticoagulation (OAC) has been shown in multiple randomized controlled studies to reduce stroke; however, risk of hemorrhage may limit applicability or patient acceptance, as has been demonstrated by frequent discontinuation of these medications (~50% of patients within 2 years of treatment initiation) [1–4].

Based on the observation that greater than 90% of cardiac thrombi have been shown to occur in the left atrial appendage (LAA) in patients with AF [5, 6], percutaneous LAA occlusion (LAAO) has been developed over the past two decades as an alternative to systemic anticoagulation. When compared to OAC using coumadin, LAAO with the Watchman™ device (Boston Scientific, Marlborough, MA) has been shown to have comparable rates of ischemic stroke and reduced rates of hemorrhagic stroke which are attributed to the use of OAC [7–11].

Despite these data, there are procedural and device-related obstacles to the adoption of the currently available LAAO devices. First, the extensive imaging required for precise sizing and placement of the currently available LAAO devices, due to the need to closely match LAA ostium diameter to device size, involves procedural transesophageal echocardiography (TEE), which requires general anesthesia. A second limitation relates to the need to ensure that the implant is delivered coaxially by orienting the delivery catheter in line with the LAA axis to provide a good seal and anchoring; however, there is limited ability to achieve optimal alignment. Moreover, many LAA ostia are oval shape and not round, and this may present a sealing challenge to current devices. Lastly, device-related procedural/periprocedural complications include pericardial effusions, the continued use of OAC postimplant as is required by product labeling in the United States, and concerns regarding device-related thrombus [12–14].

The Conformal Left Atrial Appendage Seal (CLAAS®, Conformal Medical, Inc., Nashua, NH) is designed to overcome these limitations by providing a simplified LAAO procedure. We present the early feasibility evaluation of this device in bench testing and a canine model.

## 2. Materials and Methods

### 2.1. Device Description

**Figure 1:**
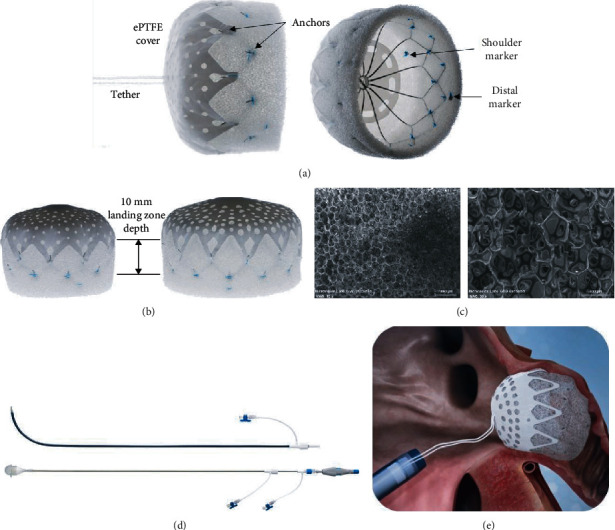
CLAAS system: (a) CLAAS regular implant with tether (left) and showing endoskeleton (right), (b) CLAAS regular (left) and large (right) implants, (c) SEM of porous polyurethane matrix material at ×15 (left) and ×50 (right) showing open cell structure, (d) CLAAS delivery system access sheath (top) and delivery catheter with loading cone (bottom), and (e) CLAAS implant positioned in LAA with tether to delivery catheter.

The CLAAS system is comprised of an implant and a custom delivery system. The implant was designed to overcome limitations of earlier LAA closure devices including the presence of residual leaks, complicated sizing procedures, placement requiring the need for TEE and general anesthesia, periprocedural complications, and device-related thrombus using only 2 implant sizes. A flexible nitinol endoskeleton combined with a porous polyurethane-carbonate matrix foam cup creates a highly conformable implant which can adapt to unique LAA anatomies, sealing against irregular projections and shapes. The foam also provides a 5 mm atraumatic distal tip (foam bumper) for procedural safety during delivery. An expanded polytetrafluoroethylene (ePTFE) surface remains after removal of the flexible tether used to connect to the delivery system (Figure 1). The design of the combined endoskeleton and foam also facilitates controlled delivery by slowing the speed of expansion. The foam, which has a highly porous surface area, promotes tissue ingrowth from the LAA (Figure 1). The endoskeleton incorporates two rows of anchors (10 per row on the regular, 12 per row on the large) for secure engagement with the LAA wall.

The implant is designed such that only two implant sizes are required to close a large range of LAA sizes, from 13 mm to 32 mm mean diameter. In addition, the implant may be successfully placed without requiring a strict coaxial angle of approach to the LAA axis during placement, simplifying the implantation procedure. The ePTFE cover provides a smooth surface facing the LA while the removable attachment tether, a high strength suture, allows that surface to be metal-free. Due to its inherently occlusive nature, the ePTFE cover has perforations that enable blood to flow at arterial pressure through the implant as mitigation should the device embolize. To enable visualization under fluoroscopy during placement, there are four platinum/iridium markers sewn onto the distal end of the inside of the foam cup and one marker located at the shoulder of the endoskeleton for alignment with the LAA ostium.

The CLAAS delivery system (Figure 1) is delivered to the LA through a long access sheath (available with single and double curve tips) introduced via a femoral venous access and a transseptal approach. Device size selection is based on mean LAA ostial diameter, with the regular designed for LAA mean diameters of 13-25 mm and the large device for mean diameters of 20-32 mm. Both implants require a minimum LAA depth of 10 mm (Figure 1). While both are fabricated using a 20 mm long foam cup, they can accommodate this short landing zone due to the short length of the endoskeleton and the unsupported distal 5 mm of foam, which creates an atraumatic “bumper” that can be collapsed against the back wall of the LAA.

The regular system fits an 18 F short venous access sheath, and the large system fits a 20 F. The implant is attached to the delivery catheter with a flexible suture tether which is used for recapture and redeployment prior to final release. The flexible tether permits the implant to sit tension-free following deployment. This differs from devices which use cable attachments that can torque the implant after deployment but prior to final release. Unlike other LAA closure devices, the CLAAS implant seals even when off axis and therefore does not require the delivery sheath to be precisely oriented coaxial to the LAA ostium (Figure 1).

### 2.2. In Vitro Conformability Assessment

A conformability assessment was made to evaluate how the implants adapt to seal LAA ostia as they transition from round to oval. Testing was conducted on both size CLAAS implants (regular and large) and on the 27 mm Watchman™ 2.5 device for comparison. The LAA was modeled utilizing an appropriately sized silicone tube submerged in a water bath at 37°C and held in a vise to squeeze the tube to make it oval. The regular CLAAS implant is designed to anchor in a LAA ostium with a mean diameter of 25 mm or less, while the large implant is designed to anchor in an ostium with a mean diameter of 33 mm or less. Therefore, a 25 mm inner diameter (ID) silicone tube was used to evaluate the regular implant and a 33 mm ID tube for the large implant. The 27 mm Watchman 2.5 device was also evaluated in the same 25 mm ID tube, and its maximum recommended LAA ostium diameter. The vise was compressed to make the silicone tube oval at approximate minor axis diameter intervals of 25, 20, 15, 10, and 5 mm for the regular CLAAS implant and the Watchman 2.5 device; 33, 25, and 20 mm for the large CLAAS implant. For each interval, the mean tube diameters were calculated as (*D*_max_ + *D*_min_)/2, and the seal was assessed visually for any gaps.

### 2.3. In Vivo (Canine) Evaluations

Canine implantations were conducted to evaluate the ability to deliver and maintain position of the CLAAS implant for 60 days. Additional analyses included gross and histological analysis of the tissue. An acute (1 hour) study was also performed in one animal to evaluate the acute seal and thrombogenicity of the implant surface. The regular size CLAAS implants were used in all animals.

The preclinical study was conducted at American Preclinical Services, LLC (Minneapolis, MN) and was approved by the Institutional Animal Care and Use Committee. Seven healthy male canines in sinus rhythm received the CLAAS device. One animal was terminated acutely for a targeted necropsy to evaluate acute thrombogenicity, and six animals were followed for 60 days post implant. The chronic animals received daily dual antiplatelet therapy of aspirin 81 mg and clopidogrel 75 mg for 45 days following implantation. After 45 days, clopidogrel was discontinued with continuation of aspirin until termination.

Implantation was performed with the animal under general anesthesia. After obtaining venous access via the right and left femoral veins, transseptal puncture was performed using standard technique under intracardiac echo (ICE) guidance. A double curve CLAAS access sheath was then tracked across the intra-atrial septum into the left atrium after which an angiographic pigtail catheter was introduced to perform LAA angiography. After removal of the pigtail catheter, the CLAAS delivery catheter was introduced, advanced to the distal end of the access sheath and deployed. A “tug test” was then performed to ensure adequate anchoring, after which sealing was documented by ICE and angiographic evaluation. If the release criteria were not met, the CLAAS implant was partially recaptured, repositioned, and redeployed.

The acute animal was maintained for one hour then euthanized, after which a targeted necropsy and device evaluation was performed to assess for device position, leaks, thrombus, perforation, and signs of embolism in downstream organs.

The chronic animals were recovered from anesthesia and maintained for approximately 60 days. TTE was performed immediately after implantation and at 2 and 45 days postimplant to assess for device position, leaks, thrombus, and pericardial effusions. At the designated time interval, animals underwent repeat TTE after which they were sacrificed, and necropsies were performed including an assessment of the downstream organs. The device was evaluated in situ and photographed, and tissues were prepared for histological assessment.

## 3. Results

### 3.1. In Vitro Conformability Evaluations

**Figure 2:**
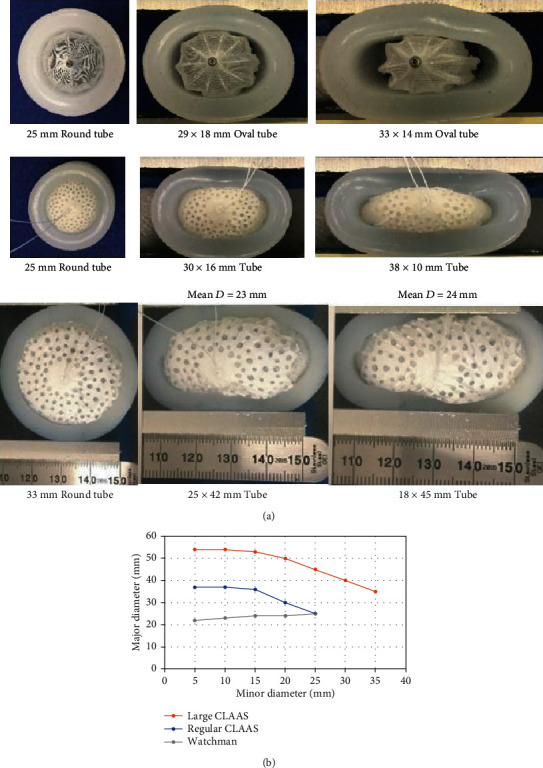
In vitro conformability testing: (a) conformability testing showing a 27 mm Watchman 2.5 (top), a 27 mm CLAAS implant (middle), and a 35 mm CLAAS implant going from round to oval and (b) graphical depiction showing response of CLAAS and Watchman 2.5 implants to compression.

Conformability outcomes of the Watchman 2.5 and CLAAS implants are shown in Figure 2.  Table 1 shows the seal at each measured point. As the CLAAS implant was compressed in the minor diameter, it increased in the major diameter thereby filling the oval space as seen in the images and demonstrated in the graphs of Figure 2. The regular CLAAS implant was able to seal round openings up to 25 mm and oval openings as eccentric as 15 × 33 mm. As the opening approached 10 × 36 mm, gaps were observed along the device edges on the major axis.

In our model, the Watchman 2.5 showed gaps even in the 25 mm diameter tube indicating that it may need to be more oversized to provide an adequate seal. The Watchman 2.5 was unable to accommodate the more oval openings as it maintains a round configuration when compressed in one direction. This is shown in Figure 2 where the major diameter decreased as the minor diameter was reduced.

As seen in Table 1, the large CLAAS implant was able to provide a complete seal in round openings up to 33 mm in diameter and oval openings down to 20 × 40 mm before gaps were observed.

### 3.2. In Vivo (Canine) Evaluations

A CLAAS implant (regular size) was successfully implanted in all seven animals. Postdeployment ICE evaluation demonstrated that CLAAS implants were appropriately positioned with good seals and without thrombus or signs of perforation in all animals. Additionally, there were no pericardial effusions. Transthoracic echocardiography performed at 2, 45, and 60 days postimplant confirmed stable device position with complete seal without leak, thrombus, or pericardial effusions in all animals.

**Figure 3:**
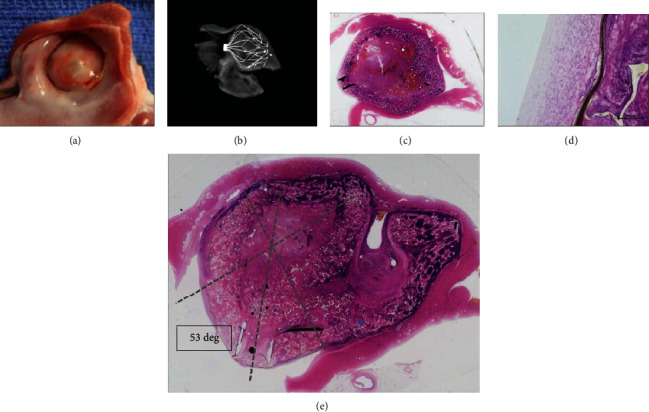
CLAAS gross and histological findings: (a) photograph of the gross appearance of the CLAAS device from the left atrium showing complete healing of the implant which was covered by a thin neointima and complete seal of the LAA ostium. (b) A/P view of endoskeleton. (c, d) Sagittal hematoxylin and eosin- (H&E-) stained sections showing complete occlusion of the LAA ostium. Also noted is the absence of thrombus or signs of inflammation. (e) H&E stain showing complete seal even with off-axis positioning of the implant. The LAA is filled with a mixture of the device material, fibrous connective tissue, and minimal residual thrombus. The ostium of the LAA is covered by thin neointima comprised of fibrous connective tissue.

Gross examination of the acute animal implant at necropsy showed the implant to be well positioned and secured, with good apposition and sealing of the LAA ostium (Figure 3). There was no visible thrombus on the surface of the implant. There was also no pericardial effusion or evidence of thromboembolism to the brain, lungs, liver, spleen, and kidneys.

All six chronic animals survived to the scheduled date of termination (62-64 days postimplant) without evidence of device-related complications or clinically significant changes in health status. Follow-up TTE imaging at 2, 45, and 60 days showed all devices to be appropriately positioned. Serial TTE and postmortem evaluations showed all devices to be well positioned, with a complete seal without thrombus. In one animal, two barbs were observed at the epicardial LAA surface without associated signs of pericardial effusion, discoloration, hemorrhage, or inflammation.

**Figure 4:**
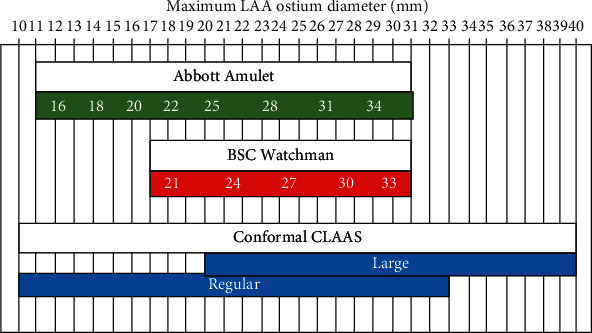
Sizing chart. Comparison of sizing for CLAAS, Watchman 2.5, and Amulet.

Histologic examination showed the atrial aspect of the implants with complete neointima covering. A representative example is shown in Figure 4. In the same figure, the LAA sealing occurs in the presence of the foam folding into itself to accommodate the irregularities of the LAA and in spite of the CLAAS implant axis being off-set at a 53-degree angle to the axis of the LAA. In the chronic animals, all the transseptal access sites had a healed appearance and were considered normal for the procedure. There was no mural thrombus present in or around the device surfaces. Minimal inflammation was noted in the tissues adjacent to the implants primarily comprised of lymphocytes, macrophages, and multinucleated giant cells. There were no abnormal findings noted in regional lymph nodes nor evidence of thromboembolism noted in selected organs.

## 4. Discussion

This study outlines the design goals and preclinical evaluations of the CLAAS system. The bench evaluations show the potential for two sizes of implants to conform to and seal a wide range of LAA sizes and shapes as compared to the Watchman 2.5 device. This device was chosen for this comparison because it was the only FDA-approved LAA closure device at the time of testing and has the largest body of randomized controlled data of any of the available LAA closure devices, making it an ideal comparison for a feasibility preclinical assessment of a novel implant. The animal explants demonstrate complete sealing of the LAA with an appropriate biological response to the implants.

The in vitro evaluations demonstrated that the CLAAS implant expands in width (the major diameter increases) as it is compressed from two sides (the minor diameter decreases), a property not observed when testing the Watchman 2.5 device. The Watchman 2.5 tends to stay round in shape while decreasing in diameter when compressed, which may prevent formation of a complete seal. Specifically, in contrast to the CLAAS, the Watchman 2.5 major diameter decreases coincident to minor diameter decreases.

The ability for an implant to expand in width during compression, as was seen with the CLAAS implant, can have several advantages in LAA closure. It may allow the implant to more effectively seal oval-shaped LAA ostia and should also allow the implant to effectively seal LAAs with a maximum diameter larger than the implant nominal diameter (IND), as long as the mean LAA diameter is smaller than the IND. This feature is unique to the CLAAS implant and is one reason that two implant sizes can effectively seal a large range of LAAs (Figure 4). Lastly, the ability of the device to conform to a noncircular ostium may allow for additional contact with ostial tissue which could also be beneficial for effective anchoring.

The CLAAS system was also evaluated in a well-accepted canine model for assessing LAAO devices [15–21]. The acute canine studies demonstrated that the device could be successfully deployed providing complete LAA seal using standard clinical techniques. The chronic animal studies demonstrated the durability of the initial closure with uniform healing with minimal inflammation. No device-related thrombi were observed in these animals, all of whom received aspirin and clopidogrel without postprocedural systemic oral anticoagulation.

Figure 3 shows the ability of the foam to fold into itself and accommodate the irregularities of the LAA and the implant to effectively seal even when placed off-axis. In this example, the CLAAS implant was placed in the LAA at a 53-degree angle to the axis of the LAA; yet, the LAA is sealed, and the CLAAS implant functioned as designed. This will simplify the implantation procedure by reducing the critical positioning of the transseptal puncture to optimally align the sheath with the axis of the LAA.

LAAO is emerging as an important alternative to oral anticoagulation for patients with AF who are at high risk for stroke, having been validated in randomized comparisons of warfarin and the Watchman device. These studies demonstrated equivalent reductions in systemic thromboembolism and stroke, fewer bleeding events, and 4-year results showing a mortality advantage with LAAO. Though the first-generation LAAO device has proven clinical utility, it nevertheless has important limitations including perforation risk, exact sizing requirements, precise delivery requiring TEE guidance, and general anesthesia. First-generation LAAO devices require predeployment LAA measurements to guide selection of the appropriate device size (Watchman 2.5 = 5 sizes, Amulet = 8 sizes) and TEE guidance to ensure coaxial deployment and to evaluate for leak. The need for continuous TEE and associated general anesthesia increases procedural risk and adds cost and logistic challenges. The design of the first-generation devices also includes thrombogenic material (polyethylene terephthalate) coverings and deployment rod attachment sites and is nonconforming (radially rigid). These design features are associated with observed leaks of up to 50% and device-related thrombus which has led to the mandate for postprocedure OAC [22].

To address these concerns, the CLAAS implant was designed to provide an improved seal, with a less traumatic, easier delivery and noncoaxial positioning, with an inherently less thrombogenic design. The CLAAS design which features an ePTFE-covered foam cup with an embedded nitinol endoskeleton and tether release mechanism was selected to achieve these goals. The combination of a foam cup coupled with a compliant endoskeleton provides a less distortive, more conforming closure than first-generation devices. The tether design eliminates the need for a threaded rod attachment site which is associated with thrombus formation [22, 23]. As a tissue scaffold, ePTFE was selected for its nonthrombogenic nature. The ultimate goal is to have the CLAAS implant delivered without the need for procedural TEE, general anesthesia, and postprocedure oral anticoagulation.

## 5. Study Limitations

The silicone model is more rigid than the LAA tissue and therefore is likely a worst-case assessment in terms of sealing of the implants. The Watchman 2.5 device is a first-generation LAA closure device; the newest version, Watchman FLX, and other currently available devices may have different results in this testing. The animal model used is limited by differences found in healing responses between young canines and elderly humans. As with previous LAA preclinical work, this model was selected due to the similarity of key LAA anatomic features and established precedence in the evaluation of LAA occlusion devices.

## 6. Conclusions

The conformability bench testing demonstrated the unique ability of the CLAAS implant to conform to and seal a wide range of oval LAA ostium sizes with just two implant sizes. The acute and chronic animal results consistently showed good seal without thrombus and documented successful off-axis closure of the LAA. A clinical study is ongoing to further demonstrate safety and feasibility in patients.

## Figures and Tables

**Table 1:** Seal testing of implants in silicone tubes.

Device	Silicone tube inner diameter (mm)	*D*_min_ × *D*_max_ (mm)	*D*_mean_ (mm)	Seal result
27 mm CLAAS	25	25 × 25	25	Sealed
27 mm CLAAS	25	20 × 30	25	Sealed
27 mm CLAAS	25	15 × 33	24	Sealed
27 mm CLAAS	25	10 × 36	23	Gaps
27 mm Watchman 2.5	25	25 × 25	25	Gaps
27 mm Watchman 2.5	25	20 × 30	25	Gaps
27 mm Watchman 2.5	25	15 × 33	24	Gaps
27 mm Watchman 2.5	25	10 × 36	23	Gaps
35 mm CLAAS	33	33 × 33	33	Sealed
35 mm CLAAS	33	30 × 35	32.5	Sealed
35 mm CLAAS	33	25 × 38	31.5	Sealed
35 mm CLAAS	33	20 × 40	30	Sealed
35 mm CLAAS	33	15 × 44	29.5	Gaps

## Data Availability

All data is in the Design History Files at Conformal Medical, Inc. (Nashua, NH, USA).

## Abbreviations

AF: Atrial fibrillation
CLAAS: Conformal Left Atrial Appendage Seal
ePTFE: Expanded polytetrafluoroethylene
ICE: Intracardiac echo
IND: Implant nominal diameter
LAA: Left atrial appendage
LAAO: Left atrial appendage occlusion
OAC: Oral anticoagulation
TEE: Transesophageal echocardiography
TTE: Transthoracic echocardiography.
